# Functional Outcomes of Early vs. Delayed Arthroscopic Repair for Traumatic and Degenerative Rotator Cuff Tears: A Retrospective Cohort Study

**DOI:** 10.3390/jcm15062205

**Published:** 2026-03-13

**Authors:** Yuzhi Chen, Yucheng Lin, Sinuo Shen, Jinge Qi, Jinan Wei, Jiachen Sun, Jun Lu

**Affiliations:** 1 School of Medicine, Southeast University, Nanjing 210000, China; cy2677044778@163.com (Y.C.); 220234359@seu.edu.cn (S.S.); 18936975749@163.com (J.Q.); 2 The Center of Joint and Sports Medicine, Orthopedics Department, Medical School, Zhongda Hospital, Southeast University, Nanjing 210000, China; linyucheng_seu@163.com (Y.L.); jinanwei@163.com (J.W.)

**Keywords:** rotator cuff tear, surgical timing, traumatic tears, degenerative tears, arthroscopic repair, functional outcomes

## Abstract

**Background/Objectives**: The optimal surgical timing for rotator cuff tears (RCTs) remains controversial, particularly regarding how tear etiology influences the final functional recovery. This study aimed to compare the clinical outcomes of early versus delayed arthroscopic repair stratified by etiology, providing evidence for etiology-specific surgical timing. **Methods**: A retrospective cohort study was conducted on 183 patients who underwent arthroscopic rotator cuff repair for isolated full-thickness supraspinatus tears. Patients were stratified into traumatic (*n* = 74) and degenerative (*n* = 109) groups based on etiology. They were further divided into early-repair and delayed-repair subgroups based on symptom duration (traumatic cut-off: 3 months; degenerative cut-off: 6 months). Clinical outcomes were assessed preoperatively and at the final follow-up using the Visual Analog Scale (VAS) for pain, American Shoulder and Elbow Surgeons (ASES) score, University of California at Los Angeles (UCLA) score, and range of motion. Complications, including retear rates and stiffness, were recorded. **Results**: In the traumatic group, early repair yielded significantly better postoperative pain relief (VAS) and higher functional scores (ASES and UCLA) compared to delayed repair. Notably, the delayed traumatic group exhibited a significantly higher retear rate compared to the early group (16.7% vs. 2.6%; *p* = 0.039). Conversely, in the degenerative group, comparisons between early and delayed repair revealed no significant differences in the final functional scores, pain levels, or complication rates (*p* > 0.05). **Conclusions**: Surgical timing significantly impacts outcomes in traumatic RCTs, where early repair is critical to optimize functional recovery and minimize retear risks. In contrast, delayed arthroscopic repair for degenerative tears yielded comparable outcomes to early repair, suggesting that an initial trial of conservative management is safe and does not compromise final surgical outcomes.

## 1. Introduction

Rotator cuff tears (RCTs) are a prevalent source of shoulder pain and disability, representing a significant burden on healthcare systems worldwide [1]. Epidemiological studies indicate that while a minority of tears are associated with acute trauma, the majority originate from degenerative processes within the shoulder joint. Consequently, distinct pathophysiological mechanisms are attributed to RCTs of these differing etiologies. Traumatic tears typically result from acute events such as falls or forceful abduction-external rotation of the arm, and are more prevalent in younger patients with relatively preserved tendon quality [2]. In contrast, degenerative RCTs are primarily associated with age-related tendon wear, characterized by a progressive pathological cascade: initiating with tendinopathy, such as disorganization of collagen fibers and mucoid degeneration, advancing to partial-thickness tears, and ultimately culminating in full-thickness tears accompanied by irreversible changes such as muscle atrophy (MA) and fatty infiltration (FI) [3,4]. These disparities in pathophysiological mechanisms suggest that the natural history, and therefore the optimal management strategy, may vary substantially between etiologies.

Arthroscopic rotator cuff repair (ARCR) is widely regarded as the gold standard for patients with failed conservative management or acute traumatic rotator cuff tears [1]. The central therapeutic philosophy of this procedure is to restore structural integrity via anatomic footprint reconstruction under low-tension conditions, thereby creating an optimal biomechanical environment for tendon-to-bone healing. While prior studies report significant functional improvements and acceptable complication rates following ARCR for isolated full-thickness [5] and partial-thickness tears [6], clinical outcomes remain variable, with retear rates reported between 10 and 40% [7]. For traumatic tears, the consensus leans heavily towards early intervention to prevent tendon retraction and halt the progression of FI and MA. Crucially, once established, these degenerative changes are considered largely irreversible, thereby compromising tendon-to-bone healing potential [8,9]. Gutman et al. [10] identified a specific functional “drop-off” when surgery for traumatic tears is delayed beyond 4 months, suggesting a finite window of opportunity to restore structural integrity before permanent biological compromise occurs. Similarly, Liu et al. [11] demonstrated that early repair (≤3 months) significantly reduces retear rates compared to delayed management in acute cases. Thus, for traumatic pathology, time is a critical predictor of structural success.

However, the influence of surgical timing on degenerative tears is far more controversial. Unlike acute injuries, degenerative tears develop over a protracted course, leading some authors to question the necessity of urgent repair. Godshaw et al. [12] and Baum et al. [13] observed that while delayed repair may involve worse baseline scores, these patients often demonstrate a “catch-up” phenomenon, ultimately achieving functional outcomes equivalent to those treated early. Furthermore, Liu et al. [14] reported that, compared with non-traumatic rotator cuff tears (RCTs), traumatic RCTs present with more severe preoperative pain and functional impairment, while postoperative clinical outcomes are comparable between the two groups.

Despite these distinctions, existing cohort studies frequently apply a uniform definition of “delayed repair” for both traumatic and degenerative RCTs [11]. However, this approach introduces methodological inconsistency: applying an acute timing threshold to a chronic disease process may oversimplify the heterogeneous biological behavior of degenerative tears. Consequently, the ideal timing of surgical intervention remains to be fully elucidated, and predictors of poor recovery have not been thoroughly investigated within the context of etiology-specific timing strategies.

To address this gap, this study aimed to evaluate the impact of surgical timing on postoperative functional outcomes and complication rates using etiology-specific timing criteria. By applying differentiated timing thresholds for traumatic and degenerative tears, this study sought to clarify whether the benefits of early repair are universal or specific to traumatic pathology, thereby providing evidence to guide individualized surgical decision-making.

## 2. Materials and Methods

### 2.1. Patient Selection

A retrospective cohort study was conducted on consecutive patients receiving arthroscopic repair for isolated full-thickness supraspinatus tears at Zhongda Hospital, Southeast University, between 1 January 2015, and 31 December 2018. The study protocol was reviewed and approved by the Institutional Ethics Committee of Zhongda Hospital, Southeast University (Approval No. 2020ZDSYLL076-P01; Approval Date: 15 April 2020). The requirement for informed consent was formally waived by the committee because the study involved no more than minimal risk to the subjects, relied exclusively on retrospective medical records, and all patient data were anonymized to protect privacy.

Patients were screened for eligibility based on strict inclusion and exclusion criteria to ensure a homogeneous cohort. The inclusion criteria were: (1) age ≥ 18 years with a minimum postoperative clinical follow-up of 6 months; (2) availability of a complete set of preoperative MRI scans for the operative shoulder; and (3) primary ARCR performed on the affected side. Patients were excluded if they presented with: (1) massive rotator cuff tears; (2) concomitant pathologies including glenohumeral osteoarthritis, shoulder instability, or fractures; (3) a history of prior surgical intervention on the ipsilateral shoulder; or (4) evidence of active or prior shoulder infection.

### 2.2. Clinical and Radiographic Assessment

**Figure 1:**
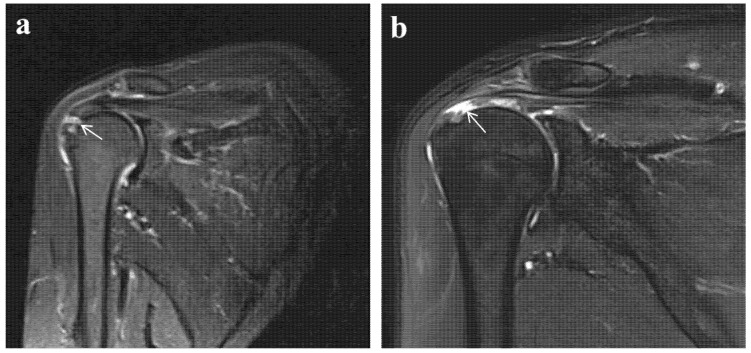
Representative MRI of traumatic and degenerative rotator cuff tear. (**a**) Oblique sagittal T2-weighted image demonstrating marked bone marrow edema within the greater tuberosity, visualized as a broad area of high signal intensity. The tear gap is filled with hyperintense fluid, and the torn tendon edge maintains a relatively preserved tension rather than appearing diffusely frayed or retracted, further supporting an acute injury pattern. (**b**) Coronal T2-weighted fat-suppressed image demonstrates a full-thickness tear of the supraspinatus tendon with significant retraction. Note the frayed, thinned, and irregular morphology of the tendon stump, characteristic of chronic degenerative changes. Note: Arrows indicate the site of the tear.

**Figure 2:**
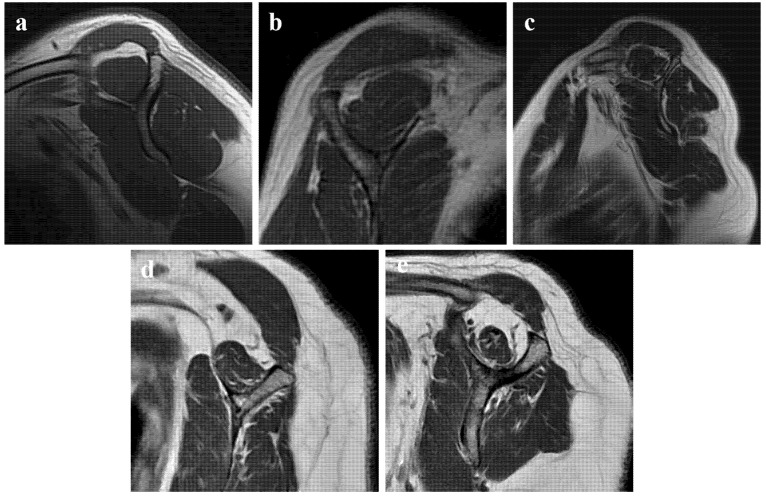
Goutallier grading of supraspinatus fatty infiltration (FI) on sagittal MRI. (**a**) Grade 0: normal muscle without fatty streaks (no FI). (**b**) Grade 1: slight FI manifested as a few fatty streaks within predominantly muscular tissue. (**c**) Grade 2: more muscle than fat; fatty areas are clearly visible but muscle still predominates. (**d**) Grade 3: approximately equal proportions of muscle and fat. (**e**) Grade 4: more fat than muscle, with severe FI and marked loss of contractile tissue.

Demographic characteristics (age, sex, BMI), comorbidities (diabetes, hypertension, smoking status), and injury details were independently extracted from electronic medical records by two investigators (J.Q. and S.S.). To ensure accuracy, all extracted data were cross-verified, and any discrepancies were resolved through consultation with a senior surgeon (J.L.). Preoperative tear characteristics were evaluated using high-resolution MRI (Figure 1). To ensure assessment reliability, two senior orthopaedic surgeons (Y.L. and J.W.) evaluated the images independently. Both reviewers were blinded to the patients’ clinical grouping and outcomes. FI of the supraspinatus was graded according to the Goutallier classification [15] (Figure 2) and muscle atrophy was assessed using the Warner classification [16]. The tangent sign was also recorded to evaluate supraspinatus muscle volume. Clinical outcomes were assessed preoperatively and at the final follow-up using the Visual Analog Scale (VAS) for pain [17], the American Shoulder and Elbow Surgeons (ASES) score [18], and the University of California, Los Angeles (UCLA) shoulder score [19].

Forward flexion (FF) and external rotation (ER) were recorded to evaluate global shoulder function. Acceptable active ROM was defined as FF > 120° and ER > 30°, representing the range of motion necessary to exclude significant functional restriction [20]. Postoperative complications, specifically retear and stiffness, were documented at the 6-month follow-up. Shoulder stiffness was defined based on the criteria by Oh et al. [20] as passive FF < 120° and/or passive ER < 30°.

### 2.3. Cohort Stratification

To evaluate the impact of etiology and timing, patients were stratified into traumatic and degenerative groups. A tear was classified as traumatic if the patient presented with: (1) a distinct history of acute trauma (e.g., traffic accident, sports injury, fall) consistent with symptom onset; and (2) a previously asymptomatic shoulder with no history of degenerative joint disease. Patients not meeting these strict criteria were classified into the degenerative group.

Subsequent stratification was performed based on the interval from symptom onset to surgery. Distinct timing thresholds were applied to reflect the differing biological nature of the tears. For the traumatic group, early repair was defined as surgery within 3 months of injury, and delayed repair as >3 months. For the degenerative group, early repair was defined as symptom duration < 6 months, and delayed repair as ≥6 months. This stratification yielded four distinct cohorts for comparative analysis.

### 2.4. Operative Technique and Rehabilitation

All surgical procedures were performed by two senior surgeons using a standardized arthroscopic protocol. Patients were placed in the beach-chair position under general anesthesia. A standard arthroscope (Smith & Nephew, Andover, MA, USA) was used for visualization. A systematic diagnostic arthroscopy was first conducted to evaluate intra-articular pathologies. Subacromial acromioplasty was performed in cases with preoperative positive impingement signs, radiographic evidence of subacromial spurs, or an intraoperatively confirmed Bigliani Type II (curved) or Type III (hooked) acromion [21,22]. This procedure aimed to eliminate subacromial impingement and create a flat acromial surface to prevent potential tendon abrasion. Prior to repair, the footprint was prepared using an arthroscopic shaver set at 5000 rpm to expose a bleeding surface. The supraspinatus tear was repaired using a single-row technique with 1–2 suture anchors (Smith & Nephew, Andover, MA, USA): a single anchor was used for small-to-medium tears (1–3 cm), while two anchors were employed for large tears (3–5 cm). The tendon was reduced and secured to ensure a tension-free repair. Management of the long head of the biceps (LHB) tendon was guided by clinical presentation and arthroscopic assessment. In cases of confirmed pathology, tenotomy was the standard intervention, whereas tenodesis (either intra-articular or subpectoral) was performed based on specific patient preferences [23].

Postoperatively, a standardized rehabilitation protocol was followed. Patients were immobilized in a shoulder abduction brace for 6 weeks. During this phase, limited passive range of motion (PROM) was permitted within a pain-free or mildly painful range. The brace was discontinued at week 7, at which point patients began training for activities of daily living (ADL), with a primary focus on restoring full range of motion and initiating early strengthening exercises. From 12 weeks postoperatively, comprehensive muscle strengthening was pursued through progressive resistance training and functional activities.

### 2.5. Statistical Analysis

Data analysis was executed using SPSS software (version 26.0; IBM Corp., Armonk, NY, USA). The normality of continuous variables was verified via the Shapiro–Wilk test, and was further confirmed through visual inspection of histograms and Q-Q plots. For normally distributed data, group comparisons were performed using independent Student’s *t*-tests, and results are presented as mean ± standard deviation (SD). Non-normally distributed variables were assessed using the Mann–Whitney U test. Categorical variables were compared using Pearson’s Chi-square test or Fisher’s exact test where appropriate. A *p* value of <0.05 was considered to indicate statistical significance.

## 3. Results

### 3.1. Patient Selection and Demographic Characteristics

**Figure 3:**
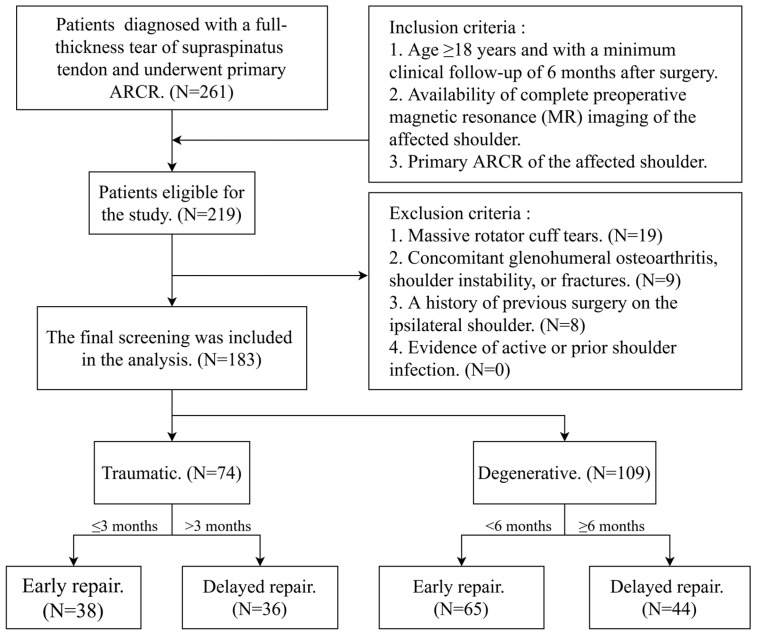
Flowchart of Patient Selection and Grouping.

A total of 183 patients met the inclusion criteria and were included in the final analysis, comprising 74 individuals in the traumatic group and 109 in the degenerative group. Based on the etiology-specific timing criteria, patients were further stratified into early-repair and delayed-repair subgroups (Figure 3).

In the traumatic cohort (*n* = 74), 38 patients underwent early repair (≤3 months) and 36 underwent delayed repair (>3 months). Baseline demographic variables, including age, sex, BMI, laterality, and smoking status, as well as systemic comorbidities and preoperative muscle quality (Goutallier and Warner grades), were statistically comparable between these two subgroups (all *p* > 0.05).

In the degenerative cohort (*n* = 109), 65 patients were treated early (<6 months) and 44 were treated in the delayed group (≥6 months). Consistent with the traumatic cohort, the early and delayed subgroups displayed similar demographic profiles and preoperative muscle status (all *p* > 0.05). The statistical comparability of baseline characteristics within both etiologic cohorts supports the validity of the direct comparative analysis of surgical timing (Table 1).

Comparison between the two etiological groups revealed distinct profiles in baseline muscle quality. Specifically, the traumatic group exhibited significantly lower grades of FI compared to the degenerative group (*p* = 0.014), with 76.2% of traumatic cases presenting as Goutallier Grade 0 or 1, compared to only 58.7% in the degenerative group. No significant differences were observed between etiologies regarding muscle atrophy (Warner Grade) or the tangent sign (Table 2).

### 3.2. Functional Outcomes and Complications

Preoperatively, functional status (VAS, ASES, UCLA, and ROM) was comparable between the early and delayed traumatic subgroups (*p* > 0.05). However, at the final follow-up, early repair yielded significantly superior clinical outcomes compared to delayed repair within the traumatic cohort.

Specifically, the early-repair group demonstrated lower mean VAS pain scores (1.39 ± 0.86 vs. 1.85 ± 0.99; *p* = 0.038) and higher mean ASES scores (83.13 ± 6.86 vs. 79.92 ± 8.04; *p* = 0.026). Similarly, postoperative UCLA scores were significantly higher in the early-repair group (29.89 ± 2.65 vs. 28.14 ± 3.91; *p* = 0.043). Regarding complications, delayed repair in traumatic cases was associated with a significantly higher retear rate (16.67% vs. 2.63%; *p* = 0.039). Although the incidence of postoperative stiffness was numerically higher in the delayed group (22.22%) compared to the early group (7.89%), this difference did not reach statistical significance (*p* = 0.083).

In contrast to the traumatic cohort, surgical timing appeared to have a negligible impact on outcomes in patients with degenerative tears. Preoperative functional scores were similar between the early and delayed subgroups. Postoperatively, functional metrics remained statistically equivalent between early and delayed repair, with no significant disparities identified in VAS pain scores (1.82 ± 0.88 vs. 1.91 ± 1.17; *p* = 0.902), ASES scores (80.28 ± 5.51 vs. 78.70 ± 8.81; *p* = 0.852), or UCLA scores (28.31 ± 3.39 vs. 27.98 ± 4.44; *p* = 0.691). Furthermore, the rates of postoperative complications, including retear (6.15% vs. 13.64%; *p* = 0.184) and stiffness (13.85% vs. 20.45%; *p* = 0.391), did not differ statistically between the timing subgroups (Table 3).

## 4. Discussion

This study aimed to evaluate the impact of surgical timing on clinical outcomes by stratifying patients based on tear etiology. By analyzing a cohort of 183 patients, our findings suggest that traumatic and degenerative tears represent distinct clinical entities that may require differentiated management strategies. The principal observation of this study is that chronological timing appears to be a stronger determinant of success for traumatic tears compared to degenerative pathology. Specifically, improved functional scores and lower retear rates were associated with early repair in the traumatic group, whereas resilience to delayed intervention was exhibited by the degenerative group.

Patients in the traumatic group who underwent early repair (≤3 months) achieved significantly better ASES and UCLA scores compared to those with delayed repair. Notably, the retear rate in the delayed traumatic group was significantly higher (16.7% vs. 2.6%; *p* = 0.039). Although some studies have reported comparable clinical results between early and delayed repair [24,25], the majority of investigators suggest that early intervention for traumatic cases is superior in reducing retear rates and optimizing functional recovery [10,11,26]. Biologically, this necessity for urgency is likely attributed to the acute retraction and rapid development of muscle atrophy that follows a sudden tendon avulsion. Gutman et al. [10] further corroborated this by identifying a functional “drop-off” when surgery for traumatic tears is delayed beyond 4 months, suggesting a finite window to restore tendon-bone continuity before irreversible changes, specifically, the replacement of contractile muscle fibers with adipose tissue and permanent muscle atrophy, which compromise the structural integrity of the repair even if the tendon is mechanically reattached [10,14]. Consequently, our data supports the consensus that traumatic tears should be prioritized for expedited surgical intervention to halt this progression [26,27].

In contrast, early repair in our degenerative cohort conferred no significant benefit. Comparisons between early (<6 months) and delayed (≥6 months) repair revealed no statistical differences in pain relief, range of motion, functional scores, or retear rates. This finding implies that degenerative tears, which likely develop over a protracted course, are often amenable to a trial of conservative management without compromising the ultimate surgical outcome. This observation is consistent with the recent literature [8,28]. Godshaw et al. [12] and Baum et al. [13] noted that while patients with chronic pathology may present with different baselines, they demonstrate a postoperative “catch-up” phenomenon, ultimately achieving satisfaction and clinical scores equivalent to those treated earlier. Similarly, no significant influence of etiology or surgical timing on final arm strength was observed by Abechain et al. [29], further corroborating that superior efficacy is not conferred by early repair in degenerative cases.

A likely explanation for this lack of time-sensitivity lies in the biological status of the muscle at presentation. Our baseline analysis revealed a significant disparity in muscle quality between the two etiologies (Table 2). The degenerative group presented with significantly higher grades of FI compared to the traumatic group (*p* = 0.014), which potentially provides a pathophysiological basis for poor postoperative recovery. In a large retrospective study of 1688 shoulders by Melis et al. [2], the development of FI was evaluated across varying tear etiologies. It was observed that FI manifests significantly earlier in traumatic tears compared to chronic onsets. Furthermore, the progression of FI was found to be accelerated particularly when the traumatic tear involved more than one tendon. While chronological time drives retraction in traumatic tears, biological degeneration dictates outcomes in chronic tears. Gladstone et al. [30] and Gerber et al. [31] have established that FI is a progressive, infiltrative process starting at the musculotendinous junction that, once established, is rarely reversed even after successful tendon reattachment. This biological irreversibility may explain the lack of functional improvement despite surgical repair. Indeed, increased retear rates and diminished functional scores have been correlated with the progression of FI in a recent meta-analysis by Wu et al. [32] and a systematic review by Khair et al. [33]. Furthermore, Chung et al. [34] and Cen et al. [35] suggest that systemic metabolic factors, such as hypercholesterolemia and obesity, exacerbate this degeneration, accelerating the “biological clock” of the muscle independent of symptom duration. Although sufficient evidence indicating that FI can be reversed by surgical repair is currently lacking, it has been reported in multiple studies that its progression is at least retarded following arthroscopic intervention [30,31,36,37]. Thus, the potential importance of surgical treatment is further underscored. Consequently, the postoperative outcomes of degenerative tears, including pain relief, functional scores, and structural integrity, were not significantly altered by varying the surgical timing. The necessity for future research to specifically quantify prognostic thresholds of FI, which may be more predictive of outcomes than chronological timing alone, is underscored by this observation.

Clinically, these findings advocate for an etiology-specific treatment algorithm. For traumatic tears, a strategy of early referral and repair is supported by the clinical data. Treating these injuries promptly, ideally within 3 months, appears beneficial in minimizing the risk of retear and optimizing functional recovery. For degenerative tears, a strict chronological deadline seems less essential. Given that established biological changes are often present and delayed surgical intervention does not appear to negatively affect postoperative outcomes, an initial trial of conservative therapy is considered a reasonable and safe option. Surgery can be reserved for patients who remain symptomatic, with the reassurance that the final result is unlikely to be negatively impacted by the delay of surgery. However, given the retrospective nature of this study and the lower level of evidence, this proposed algorithm should be applied with caution and tailored to individual patient factors.

Our study is not without limitations. First, potential selection bias is inherent to the retrospective cohort design. As a single-center study, our findings reflect the specific patient population and surgical practices of our institution and may not be fully generalizable to broader demographics. Second, the follow-up duration was relatively limited. To address this limitation, continuous surveillance of the current cohort is being maintained to evaluate mid- to long-term outcomes and to validate the durability of the observed results. Third, statistical robustness was constrained by the relatively small sample size (*n* = 183) and the exploratory decision not to adjust for multiple comparisons to avoid Type II errors. Consequently, subtle differences in secondary outcomes may have been missed, and marginally significant findings warrant cautious interpretation. Finally, our cohort was limited to isolated supraspinatus tears, and findings should not be extrapolated to massive or multi-tendon involvements.

## 5. Conclusions

In this retrospective cohort study, traumatic and degenerative rotator cuff tears appear to follow distinct recovery trajectories. Our findings indicate that for traumatic tears, significantly optimized functional recovery and a reduction in retear rates were observed following early repair, a trend likely attributable to the mitigation of irreversible structural changes via surgical reconstruction. In contrast, degenerative tears demonstrated comparable clinical results regardless of surgical timing, suggesting that an initial trial of conservative management is a reasonable option. These findings advocate for an etiology-specific surgical strategy. Future large-scale, prospective studies involving long-term imaging surveillance are warranted to investigate the efficacy of surgery in retarding or reversing tissue degeneration across different etiologies.

## Figures and Tables

**Table 1:** Baseline characteristics of patients with traumatic and degenerative RCTs in the early- and delayed-repair groups.

	Early-Repair Group	Delayed-Repair Group	*p* Value
**Traumatic, *n* = 74**			
Sex			0.671
Male, *n* (%)	14 (36.84)	15 (41.67)	
Female, *n* (%)	24 (63.16)	21 (58.33)	
Age, years	52.21 ± 6.92	55.75 ± 7.19	0.066
BMI	25.11 ± 2.90	25.59 ± 2.40	0.330
Involved side			0.405
Left shoulder, *n* (%)	14 (36.84)	10 (27.78)	
Right shoulder, *n* (%)	24 (63.16)	26 (72.22)	
Tobacco use, *n* (%)	6 (15.79)	5 (13.89)	0.818
Diabetes, *n* (%)	4 (10.53)	5 (13.89)	0.658
Hypertension, *n* (%)	12 (31.58)	13 (36.11)	0.680
Fatty infiltration			0.211
Early stage, *n* (%)	32 (84.21)	26 (72.22)	
Advanced stage, *n* (%)	6 (15.79)	10 (27.78)	
Muscle atrophy			0.637
Early stage, *n* (%)	35 (90.48)	32 (88.89)	
Advanced stage, *n* (%)	3 (9.52)	4 (11.11)	
**Degenerative, *n* = 109**			
Sex			0.433
Male, *n* (%)	27 (41.54)	13 (29.54)	
Female, *n* (%)	38 (58.46)	31 (70.46)	
Age, years	54.42 ± 7.85	55.73 ± 8.36	0.301
BMI	25.40 ± 2.66	25.78 ± 2.75	0.417
Involved side			0.716
Left shoulder, *n* (%)	20 (30.77)	15 (34.09)	
Right shoulder, *n* (%)	45 (69.23)	29 (65.91)	
Tobacco use, *n* (%)	6 (9.23)	6 (13.67)	0.471
Diabetes, *n* (%)	9 (13.85)	6 (13.67)	0.975
Hypertension, *n* (%)	23 (35.38)	17 (38.67)	0.730
Fatty infiltration			0.261
Early stage, *n* (%)	41 (63.08)	23 (52.27)	
Advanced stage, *n* (%)	24 (36.92)	21 (47.73)	
Muscle atrophy			0.087
Early stage, *n* (%)	57 (87.69)	33 (75.00)	
Advanced stage, *n* (%)	8 (12.31)	11 (25.00)	

BMI: body mass index.

**Table 2:** Muscle quality of the supraspinatus in patients with traumatic and degenerative RCTs.

	Traumatic, *n* = 74	Degenerative, *n* = 109	*p* Value
**Goutallier Grade** **, *n* (%)**			**0.014**
0	29 (39.19)	20 (18.35)	
1	29 (39.19)	44 (40.37)	
2	9 (12.16)	21 (19.26)	
3	4 (5.41)	15 (13.76)	
4	3 (4.05)	9 (8.26)	
**Warner Grade, *n* (%)**			0.143
0	44 (59.46)	46 (42.20)	
1	23 (31.08)	44 (40.37)	
2	5 (6.76)	14 (12.84)	
3	2 (2.70)	5 (4.59)	
**Tangent sign of the supraspinatus, *n* (%)**			0.130
Positive	7 (9.46)	19 (17.43)	
Negative	67 (90.54)	90 (82.57)	

Goutallier Grade: Grade 0: No fatty infiltration; Grade 1: Some fatty streaks; Grade 2: More muscle than fat; Grade 3: Equal amounts of muscle and fat; Grade 4: More fat than muscle. Warner Grade: Grade 0: No atrophy; Grade 1: Mild atrophy; Grade 2: Moderate atrophy; Grade 3: Severe atrophy. Tangent sign of the supraspinatus: If the supraspinatus muscle belly lies entirely below this tangent line, the sign is considered positive. Otherwise, it is considered negative. Statistically significant values (*p* < 0.05) are highlighted in bold.

**Table 3:** Preoperative and postoperative results of patients with traumatic and degenerative RCT in the early- and delayed-repair groups.

	Preoperative			Postoperative		
	Early-Repair Group	Delayed-Repair Group	*p* Value	Early-Repair Group	Delayed-Repair Group	*p* Value
**Traumatic, *n* = 74**						
VAS score	4.89 ± 0.77	4.76 ± 0.67	0.442	1.39 ± 0.86	1.85 ± 0.99	**0.038**
ASES score	40.11 ± 4.79	39.69 ± 4.50	0.668	83.13 ± 6.86	79.92 ± 8.04	**0.026**
UCLA score	13.97 ± 1.92	13.36 ± 1.69	0.080	29.89 ± 2.65	28.14 ± 3.91	**0.043**
Patients with ROM FF > 120°, *n* (%)	9 (23.68)	11 (30.56)	0.658	32 (84.21)	26 (72.22)	0.211
Patients with ROM ER > 30°, *n* (%)	12 (31.58)	14 (38.89)	0.530	34 (89.47)	27 (75.00)	0.102
Patients with joint stiffness, *n* (%)				3 (7.89)	8 (22.22)	0.083
Patients with rotator cuff retear, *n* (%)				1 (2.63)	6 (16.67)	**0.039**
**Degenerative, *n* = 109**						
VAS score	4.56 ± 0.64	4.67 ± 0.67	0.390	1.82 ± 0.88	1.91 ± 1.17	0.902
ASES score	46.43 ± 5.07	48.34 ± 5.32	0.060	80.28 ± 5.51	78.70 ± 8.81	0.852
UCLA score	16.03 ± 1.85	16.41 ± 1.78	0.305	28.31 ± 3.39	27.98 ± 4.44	0.691
Patients with ROM FF > 120°, *n* (%)	15 (23.08)	10 (22.73)	0.966	46 (70.70)	30 (68.18)	0.773
Patients with ROM ER > 30°, *n* (%)	17 (26.15)	13 (29.54)	0.697	49 (75.38)	32 (70.45)	0.755
Patients with joint stiffness, *n* (%)				9 (13.85)	9 (20.45)	0.391
Patients with rotator cuff retear, *n* (%)				4 (6.15)	6 (13.64)	0.184

VAS, visual analogue scale; ASES, American shoulder and elbow surgeons; UCLA, The University of California, Los Angeles; ROM, range of motion; FF, forward flexion; ER, external rotation. Statistically significant values (*p* < 0.05) are highlighted in bold.

## Data Availability

The datasets used and analyzed during the current study are available from the corresponding author on reasonable request.

## Abbreviations

The following abbreviations are used in this manuscript:

ARCR	Arthroscopic rotator cuff repair
ASES	American Shoulder and Elbow Surgeons score
BMI	Body mass index
FI	Fatty infiltration
MRI	Magnetic resonance imaging
RCT	Rotator cuff tear
ROM	Range of motion
SD	Standard deviation
SPSS	Statistical Package for the Social Sciences
T1WI	T1-weighted imaging
T2WI	T2-weighted imaging
UCLA	University of California, Los Angeles shoulder score
VAS	Visual analogue scale
